# Changes in Patient Experiences of Hospital Care During the COVID-19 Pandemic

**DOI:** 10.1001/jamahealthforum.2023.2766

**Published:** 2023-08-25

**Authors:** Marc N. Elliott, Megan K. Beckett, Christopher W. Cohea, William G. Lehrman, Paul D. Cleary, Laura A. Giordano, Chelsea Russ, Elizabeth H. Goldstein, Lee A. Fleisher

**Affiliations:** 1 RAND Corporation, Santa Monica, California; 2 Health Services Advisory Group, Phoenix, Arizona; 3 US Centers for Medicare & Medicaid Services, Baltimore, Maryland; 4 Yale School of Public Health, New Haven, Connecticut

## Abstract

**Importance:**

It is important to assess how the COVID-19 pandemic was adversely associated with patients’ care experiences.

**Objective:**

To describe differences in 2020 to 2021 patient experiences from what would have been expected from prepandemic (2018-2019) trends and assess correlates of changes across hospitals.

**Design, Setting, and Participants:**

This cohort study compared 2020 to 2021 data with 2018 to 2019 data from 3 900 887 HCAHPS respondents discharged from 3381 HCAHPS-participating US hospitals. The data were analyzed from 2022 to 2023.

**Main Outcomes and Measures:**

The primary outcome was an HCAHPS summary score (HCAHPS-SS), which averaged 10 HCAHPS measures. The primary analysis estimated whether HCAHPS scores from patients discharged from 2020 to 2021 differed from scores that would be expected based on quarterly and linear trends from 2018 to 2019 discharges. Secondary analyses stratified hospitals by prepandemic overall star ratings and staffing levels.

**Results:**

Of the 3 900 887 HCAHPS 2020 to 2021 respondents, 59% were age 65 years or older, and 35% (11%) were in the surgical (maternity) service lines. Compared with trends expected based on prepandemic (2018-2019) data, HCAHPS-SS was 1.2 percentage points (pp) lower for quarter (Q) 2/2020 discharges and −1.9 to −2.0 pp for Q3/2020 to Q1/2021, which then declined to −3.6 pp by Q4/2021. The most affected measures (Q4/2021) were staff responsiveness (−5.6 pp) and cleanliness (−4.9 pp); the least affected were discharge information (−1.6 pp) and quietness (−1.8 pp). Overall rating and hospital recommendation measures initially exhibited smaller-than-average decreases, but then fell as much as the more specific experience measures by Q2/2021. Quietness did not decline until Q2/2021. The HCAHPS-SS fell most for hospitals with the lowest prepandemic staffing levels; hospitals with bottom-quartile staffing showed the largest decrements, whereas top-quartile hospitals showed smaller decrements in most quarters. Hospitals with better overall prepandemic quality showed consistently smaller HCAHPS-SS drops, with effects for 5-star hospitals about 25% smaller than for 1-star and 2-star hospitals.

**Conclusions and Relevance:**

The results of this cohort study of HCAHPS-participating hospitals found that patient experience scores declined during 2020 to 2021. By Q4/2021, the HCAHPS-SS was 3.6 pp lower than would have been expected, a medium effect size. The most affected measures (staff responsiveness and cleanliness) showed large effect sizes, possibly reflecting high illness-associated hospital workforce absenteeism. Hospitals that were lower performing and less staffed prepandemic may have been less resilient to reduced staff availability and other pandemic-associated challenges. However, by Q4/2021, even prepandemic high-performing hospitals had similar declines.

## Key Points

**Question:**

Did patient hospital experiences change during the COVID-19 pandemic and, if so, did the changes differ across hospitals with different patient-staff ratios and prepandemic quality (hospital overall star rating)?

**Findings:**

In this cohort study of 3 900 887 respondents of the Hospital Consumer Assessment of Healthcare Providers and Systems (HCAHPS) discharged from 2020 to 2021, comparisons of HCAHPS scores for patients with prepandemic data indicated that all HCAHPS scores declined during 2020 to 2021, especially for hospitals with lower prepandemic staffing and performance. Declines were largest for staff responsiveness and cleanliness.

**Meaning:**

The results of this study suggest that higher-staffed and higher-performing hospitals were more resilient to the conditions of the COVID-19 pandemic, but by late 2021, patients’ experience of care had declined in all hospitals.

## Teaser

This cohort study examines Hospital Consumer Assessment of Healthcare Providers and Systems survey responses from 2018 to 2019 and 2020 to 2021 to assess differences in patient experiences before and during the COVID-19 pandemic.

## Introduction

Since 2008, Hospital Consumer Assessment of Healthcare Providers and Systems (HCAHPS) survey results from patients at short-term acute care hospitals have been publicly reported with quarterly updates on the US Centers for Medicare & Medicaid Services’ (CMS) Care Compare (formerly Hospital Compare) website and have been included in the Hospital Value-Based Purchasing Program payment formula since 2013.^1^ Hospital CAHPS provides a consistent and detailed source of information for monitoring changes in patient experiences in response to changes in circumstances or policies. During the COVID-19 pandemic, hospitals suddenly faced extraordinary demands,^2^ and before the availability of vaccines, health care professionals feared being exposed to COVID-19 at work and infecting family members. To comply with US Centers for Disease Control and Prevention guidelines associated with COVID-19 infection control, hospitals implemented new protocols involving social distancing, personal protective equipment (PPE) requirements of masks and face shields, and restricted visitor access, factors that were subsequently found to be associated with adverse patient outcomes.^3^ High rates of emotional exhaustion among health care workers have been attributed to staffing shortages, uncertainty around evolving hospital protocols, unreliable PPE supplies, and incivility directed toward health care workers.^4^ Limited information has suggested that emotional exhaustion increased greatly among staff, with nearly half of nurses in 1 annual survey reporting exhaustion during 2021 and 2022.^4^ At times, hospitals became overwhelmed as admissions spiked.^5^

Early evidence suggests that these COVID-19–related changes were negatively associated with health care quality. Enhanced isolation practices and PPE requirements are thought to have been associated with a reduced focus on routine infection control practices. After years of steady declines, increases in rates of health care–acquired infections were associated with COVID-19 surges.^6^ The demands that the COVID-19 pandemic imposed on hospitals and feelings of isolation associated with restrictive family visitation policies may have disadvantaged patients. Prepandemic research suggests that compared with nonisolated patients, patients who are isolated for infection control consistently exhibit more depression and anxiety, experience more adverse events,^7^ and report worse experiences of care on HCAHPS measures.^8^ Several other potential negative associations of the pandemic protocols, including substantial delays in services, challenges communicating with physicians and nurses, and poor patient education, were identified in a single-hospital study of patients with and without COVID-19.^9^

In this study, we used cross-sectional data from US hospitals to describe how HCAHPS scores during a peak period of the COVID-19 pandemic differed from what would have been expected based on prepandemic trends. We also assessed whether patients treated in hospitals with higher prepandemic staffing levels and a better quality of care prepandemic had smaller decreases in reported experience after the start of the pandemic. We expected that the pandemic would be associated with lower HCAHPS scores, especially in hospitals with lower prepandemic staffing levels and quality of care, because such hospitals would have fewer resources and processes in place to maintain positive patient experiences.

## Methods

### Sample

The analyses presented in this article used data for all quarters (Qs) from 2018 to 2021 from the 3381 hospitals that submitted to CMS at least 25 completed HCAHPS surveys from discharged patients during at least 1 quarter during 2020 and 2021 (eMethods 1 in Supplement 1). This cohort study was approved by the institutional review board at Rand Corporation; the requirement for written informed consent was waived because only deidentified data were obtained. We followed the Strengthening the Reporting of Observational Studies in Epidemiology (STROBE) reporting guidelines.^10^

### HCAHPS Measures

The study’s primary outcome was the HCAHPS summary score (HCAHPS-SS), the average of 10 HCAHPS measures: 6 multi-item composite measures that were each given a weight of 1.0 (communication with nurses, communication with doctors, staff responsiveness, communication about medicines, care transition, and discharge information) and 4 single-item measures that were each given a weight of 0.5 (overall hospital rating, hospital recommendation, cleanliness, and quietness). Survey response options were “never,” “sometimes,” “usually,” or “always” for all measures other than discharge information (options were “yes” or “no”), care transition (options were “strongly disagree,” “disagree,” “agree,” or “strongly agree”), hospital recommendation (options were “definitely no,” “probably no,” “probably yes,” or “definitely yes”), and overall hospital rating (response options ranged from 0 [“worst possible hospital”] to 10 [“best possible hospital”]). Top-box scoring, which is the proportion of most-positive category responses (except for the overall hospital rating, for which 9 or 10 correspond to a top-box response), was used for all HCAHPS measures and the HCAHPS-SS. Secondary analyses examined each of the 10 constituent HCAHPS measures individually. The HCAHPS items and details of survey implementation protocols can be found at http://www.hcahpsonline.org.

### Other Measures

For an indicator of overall prepandemic hospital quality, we used the 2019 hospital overall star rating from CMS,^11^ which is based on 5 groups of measures associated with health care quality: mortality, readmissions, safety, patient experience (each group weighted 22%), and timely and effective care (weighted 12%). These star ratings were classified as 1 to 2 (pooled because of low frequency), 3, 4, 5, or missing.

Prior research has shown that nurse communication is the HCAHPS measure most strongly associated with overall HCAHPS ratings and recommendations^12,13^ and that nurse staffing levels are strongly associated with HCAHPS scores.^12,13,14,15,16^ Staffing level, from the 2019 American Hospital Association data, was defined as the number of full-time–equivalent nurses, registered nurses, and licensed practical nurses working in the hospital (excluding nursing home and long-term care unit staff) per 1000 adjusted patient days. We used a mean imputation for missing data (<1%) on nursing levels. Staffing levels were then classified into quartiles.

### Analyses

To make results comparable across hospitals and over time, HCAHPS data were adjusted for the mode of survey administration and patient characteristics not within the control of the hospital that were known to affect survey responses.^17,18^ To evaluate the post-2019 departure of HCAHPS-SS from prepandemic trends, we estimated 8 adjusted linear mixed-effect regression models, each of which included 9 quarters of patient-level data. Each model included data from the 8 quarters that immediately preceded the pandemic (Q1/2018 to Q4/2019) to evaluate HCAHPS-SS in 1 quarter during the pandemic period (Q1/2020 to Q4/2021). Each linear mixed-effect regression model included patient-mix variables, an indicator of the mode of survey administration, hospital random effects, a linear time trend (number of quarters since Q1/2018), 3 quarter intercepts (indicators for each of Q2, Q3, and Q4, regardless of year) to control for seasonal trends, an indicator for hospitals voluntarily submitting HCAHPS data when reporting was optional (Q1/2020 and/or Q2/2020),^19^ and a post-2019 indicator (an indicator of being in a COVID-19–era quarter during 2020 or 2021). The last term estimated the departure of the post-2019 quarter in question from the season and linear trend. Each model included hospitals with 25 completed surveys during the specified post-2019 quarter and all data from that quarter and other quarters from 2018 to 2019 for which the hospital had 25 completed surveys. We repeated these analyses for each HCAHPS measure.

In addition to overall changes, we assessed whether hospital characteristics were associated with differential changes in HCAHPS scores by stratifying by hospital characteristics 1 at a time (2019 hospital overall star rating [1-2, 3, 4, 5, and no star] and quartiles of staffing levels) and graphically illustrating trends in changes in HCAHPS-SS by hospital characteristics. We also examined variation in estimated pandemic effects across the 9 US Census Divisions. We ran sensitivity analyses described in eMethods 3 in Supplement 1 to assess whether our use of varying cohorts of hospitals was associated with the study results and whether patterns in patient volume may have accounted for the observed trends. Analyses were conducted using SAS, version 9.4 (SAS Institute), and statistical significance was set at *P *< .05.

## Results

For the 3381 hospitals used in the primary analysis, HCAHPS response rates were 25%, 25%, 24%, and 22%, respectively, for 2018, 2019, 2020, and 2021,^20^ resulting in 5 298 431 completed surveys during 2018 and 2019 and 3 900 887 completed surveys from 2020 to 2021. Of the 2020 to 2021 (2018-2019) respondents, 26% (26%) were in fair or poor self-rated health, 59% (57%) were 65 years or older, 35% (38%) were in the surgical service line, and 11% (11%) were in the maternity service line (eTables 1 and 2 in Supplement 1 show patient and hospital characteristics, respectively).

### Changes in HCAHPS Scores

**Figure 1.**
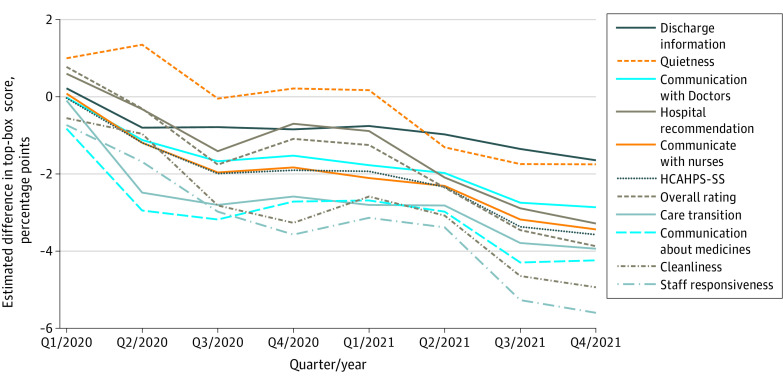
Quarterly Estimates of the Association of the COVID-19 Pandemic With Hospital Consumer Assessment of Healthcare Providers and Systems (HCAHPS) Scores by Measure A plot of the departures of HCAHPS top-box measure scores from what would have been expected had 2018 to 2019 linear and quarterly trends continued, adjusting for patient mix and mode of survey administration. Estimated quarterly pandemic effects by measure are shown. See Table 1 and eTable 1 in Supplement 1. HCAHPS-SS indicates Hospital Consumer Assessment of Healthcare Providers and Systems summary score.

The first row of Table 1 and the dark blue dotted line in Figure 1 present the estimated differences of HCAHPS-SS scores from 2020 to 2021 from expected scores based on prepandemic trends. The HCAHPS-SS was not significantly different than expected based on prepandemic trends for Q1/2020 discharges but was 1.2 percentage points (pp) lower than expected (−1.2 pp) for Q2/2020 discharges and −1.9 to −2.0 pp for Q3/2020 to Q1/2021 and then steadily worse to −3.6 pp by Q4/2021. Figure 1 and eTable 3 in Supplement 1 show comparable analyses for specific HCAHPS measures; eFigure 1 in Supplement 1 shows simple HCAHPS-SS trends by quarter from Q1/2018 through Q4/2021.

**Table 1.** Mixed-Effect Models of Adjusted HCAHPS-SS Using Independent Time Variables^a^

2020	Q1/2020 (n = 5 861 470 patients; n = 1648 hospitals)		Q2/2020 (n = 5 807 876 patients; n = 1737 hospitals)		Q3/2020 (n = 6 118 270 patients; n = 3224 hospitals)		Q4/2020 (n = 6 104 234 patients; n = 3295 hospitals)	
	Est (SE), %	*P* value	Est (SE), %	*P* value	Est (SE), %	*P* value	Est (SE), %	*P* value
Post-2019 indicator	−0.04 (0.05)	.52	−1.20 (0.06)	<.001	−2.00 (0.05)	<.001	−1.91 (0.05)	<.001
Time, linear mo	0.08 (0.01)	<.001	0.08 (0.01)	<.001	0.08 (0.01)	<.001	0.08 (0.01)	<.001
Q2 indicator	0.42 (0.03)	<.001	0.42 (0.03)	<.001	0.42 (0.03)	<.001	0.42 (0.03)	<.001
Q3 indicator	0.35 (0.03)	<.001	0.35 (0.03)	<.001	0.35 (0.03)	<.001	0.35 (0.03)	<.001
Q4 indicator	0.04 (0.03)	.19	0.04 (0.03)	.21	0.04 (0.03)	.26	0.04 (0.03)	.22
**2021**	**Q1/2021 (n = 6 099 727 patients; n = 3219 hospitals)**		**Q2/2021 (n = 6 109 713 patients; n = 3181 hospitals)**		**Q3/2021 (n = 6 084 396 patients; n = 3232 hospitals)**		**Q4/2021 (n = 5 990 289 patients; n = 3137 hospitals)**	
Post-2019 indicator	−1.93 (0.06)	<.001	−2.34 (0.06)	<.001	−3.36 (0.06)	<.001	−3.57 (0.06)	<.001
Time, linear mo	0.08 (0.01)	<.001	0.08 (0.01)	<.001	0.08 (0.01)	<.001	0.08 (0.01)	<.001
Q2 indicator	0.42 (0.03)	<.001	0.42 (0.03)	<.001	0.42 (0.03)	<.001	0.42 (0.03)	<.001
Q3 indicator	0.35 (0.03)	<.001	0.35 (0.03)	<.001	0.35 (0.03)	<.001	0.35 (0.03)	<.001
Q4 indicator	0.04 (0.03)	.27	0.04 (0.03)	.25	0.04 (0.03)	.24	0.04 (0.03)	.23

Abbreviations: est, estimation; HCAHPS-SS, Hospital Consumer Assessment of Healthcare Providers and Systems summary score; Q, quarter.

^a^ Each model includes Q1/2018 to Q4/2019 discharges, plus 1 post-2019 quarter (each model had 9 quarters). Each model was limited to hospitals with at least 25 completed surveys in their post-2019 quarter and included data from all quarters for which these hospitals had at least 25 completed surveys. Models also included hospital random effects.

By Q4/2021, 2 years into the COVID-19 pandemic, the most affected HCAHPS measures, with large^21^ departures from prepandemic trends, were staff responsiveness (−5.6 pp) and cleanliness (−4.9 pp), while communication about medicines (−4.2 pp), care transition (−3.9 pp), overall hospital rating (−3.9 pp), communication with nurses (−3.4 pp), hospital recommendation (−3.3 pp), and communication with doctors (−2.9 pp) were moderately^21^ affected. The least affected measures were discharge information (−1.6 pp) and quietness (−1.8 pp), neither of which exceeded a full top-box point less than what was expected until Q2/2021 or Q3/2021. The HCAHPS global measures, overall rating and hospital recommendation, had smaller-than-average initial declines but fell as much as the more specific measures by Q2/2021.

Rows 2 to 5 of Table 1 show the prepandemic linear trend (a small increase of approximately 1 pp per year) and the seasonal trends (scores were approximately 0.4 points higher April-September [Q2-Q3] than October-March [Q4-Q1]). Accounting for these trends allowed for a more accurate estimation of the departures of 2020 to 2021 scores from prepandemic trends.

### Variation by Hospital Type

**Figure 2.**
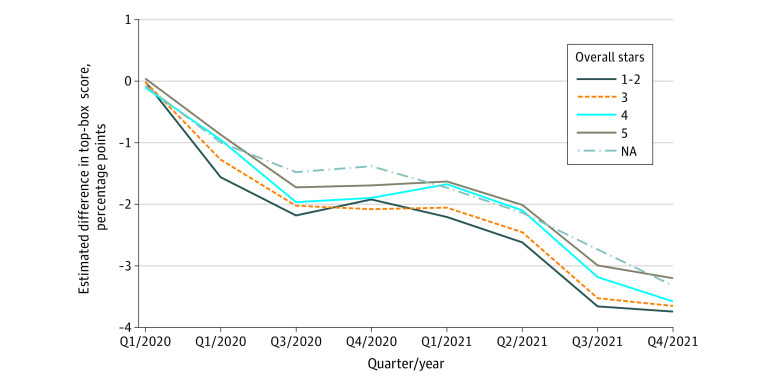
Quarterly Estimates of the Association of the COVID-19 Pandemic With the Hospital Consumer Assessment of Healthcare Providers and Systems (HCAHPS) Summary Score by 2019 Overall Stars A plot of the departures of the HCAHPS top-box summary score from what would have been expected had 2018 to 2019 linear and quarterly trends continued, adjusting for patient mix and mode of survey administration, stratified by 2019 overall star ratings. Estimated quarterly pandemic effects are shown as stratified by overall stars in 2019. See Table 2.

As shown in Table 2 and Figure 2, HCAHPS-SS fell the most for hospitals with the lowest prepandemic staffing levels: hospitals with bottom-quartile (lowest) staffing experienced the largest decrements, while top-quartile (highest) hospitals had smaller decrements during most quarters. However, by Q4/2021, the hospitals with the highest prepandemic staffing had similar decrements as the hospitals with the lowest prepandemic staffing (with a range −3.5 to −3.7 pp across groups).

**Table 2.** Estimated Association of the COVID-19 Pandemic With HCAHPS-SS in Models as Stratified by Hospital Characteristics^a^

2020	Q1/2020 (n = 5 861 470 patients; n = 1648 hospitals)		Q2/2020 (n = 5 807 876 patients; n = 1737 hospitals)		Q3/2020 (n = 6 118 270 patients; n = 3224 hospitals)		Q4/2020 (n = 6 104 234 patients; n = 3295 hospitals)	
	Est (SE), %	*P* value	Est (SE), %	*P* value	Est (SE), %	*P* value	Est (SE), %	*P* value
**Prepandemic (2019) overall star rating**								
Overall star rating, 1-2	−0.01 (0.11)	.93	−1.56 (0.11)	<.001	−2.18 (0.09)	<.001	−1.92 (0.09)	<.001
Overall star rating, 3	−0.01 (0.11)	.92	−1.28 (0.11)	<.001	−2.02 (0.09)	<.001	−2.08 (0.10)	<.001
Overall star rating, 4	−0.11 (0.10)	.29	−0.95 (0.10)	<.001	−1.97 (0.09)	<.001	−1.90 (0.09)	<.001
Overall star rating, 5	0.04 (0.13)	.76	−0.87 (0.14)	<.001	−1.73 (0.12)	<.001	−1.70 (0.12)	<.001
NA stars	−0.08 (0.26)	.78	−1.00 (0.26)	<.001	−1.48 (0.22)	<.001	−1.38 (0.22)	<.001
**2021**	**Q1/2021 (n = 6 099 727 patients; n = 3219 hospitals)**		**Q2/2021 (n = 6 109 713 patients; n = 3181 hospitals)**		**Q3/2021 (n = 6 084 396 patients; n = 3232 hospitals)**		**Q4/2021 (n = 5 990 289 patients; n = 3137 hospitals)**	
Overall star rating, 1-2	−2.21 (0.12)	<.001	−2.62 (0.12)	<.001	−3.66 (0.12)	<.001	−3.74 (0.13)	<.001
Overall star rating, 3	−2.06 (0.12)	<.001	−2.46 (0.12)	<.001	−3.52 (0.12)	<.001	−3.65 (0.13)	<.001
Overall star rating, 4	−1.67 (0.12)	<.001	−2.10 (0.12)	<.001	−3.18 (0.12)	<.001	−3.58 (0.12%)	<.001
Overall star rating, 5	−1.63 (0.16)	<.001	−2.01 (0.16)	<.001	−3.00 (0.16)	<.001	−3.20 (0.17)	<.001
NA stars	−1.73 (0.29)	<.001	−2.13 (0.29)	<.001	−2.74 (0.29)	<.001	−3.32 (0.30)	<.001
**2020**	**Q1/2020 (n = 5 861 470 patients; n = 1648 hospitals)**		**Q2/2020 (n = 5 807 876 patients; n = 1737 hospitals)**		**Q3/2020 (n = 6 118 270 patients; n = 3224 hospitals)**		**Q4/2021 (n = 5 990 289 patients; n = 3295 hospitals)**	
**Prepandemic (2019) staffing levels**								
Q1 (lowest staffing)	−0.01 (0.12)	.90	−1.83 (0.13)	<.001	−2.27 (0.10)	<.001	−2.12 (0.11)	<.001
Q2	0.05 (0.09)	.61	−1.14 (0.10)	<.001	−2.08 (0.08)	<.001	−1.98 (0.08%)	<.001
Q3	−0.17 (0.10)	.10	−0.95 (0.10)	<.001	−1.82 (0.09)	<.001	−1.73 (0.09)	<.001
Q4 (highest staffing)	0.06 (0.15)	.69	−0.98 (0.16)	<.001	−1.63 (0.13)	<.001	−1.72 (0.13)	<.001
**2021**	**Q1/2021 (n = 6 099 727 patients; n = 3219 hospitals)**		**Q2/2021 (n = 6 109 713 patients; n = 3181 hospitals)**		**Q3/2021 (n = 6 084 396 patients; n = 3232 hospitals)**		**Q4/2021 (n = 5 990 289 patients; n = 3137 hospitals)**	
Q1 (lowest staffing)	−2.26 (0.13)	<.001	−2.65 (0.13)	<.001	−3.77 (0.14)	<.001	−3.74 (0.14)	<.001
Q2	−1.94 (0.10)	<.001	−2.39 (0.10)	<.001	−3.37 (0.10)	<.001	−3.49 (0.11)	<.001
Q3	−1.86 (0.11)	<.001	−2.12 (0.12)	<.001	−3.22 (0.12)	<.001	−3.54 (0.12)	<.001
Q4 (highest staffing)	−1.44 (0.18)	<.001	−2.09 (0.18)	<.001	−2.94 (0.18)	<.001	−3.62 (0.18)	<.001

Abbreviations: est, estimation; HCAHPS-SS, Hospital Consumer Assessment of Healthcare Providers and Systems summary score; NA, not applicable; Q, quarter.

^a^ Each model included Q1/2018 to Q4/2019 discharges, plus 1 post-2019 quarter (each model had 9 quarters). Each model was limited to hospitals with at least 25 completed surveys in their post-2019 quarter and included data from all quarters for which these hospitals had at least 25 completed surveys. Models also included hospital random effects.

**Figure 3.**
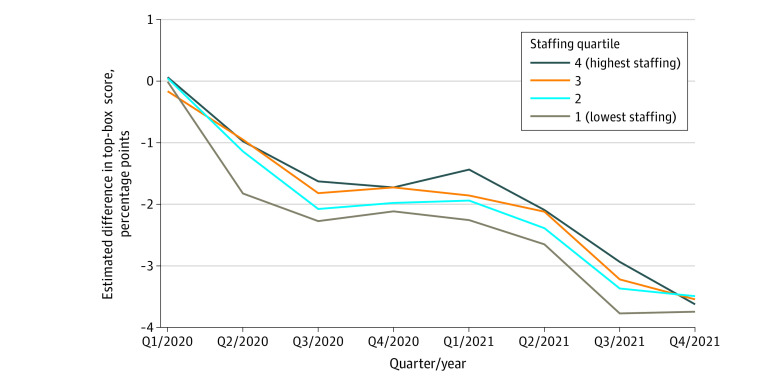
Quarterly Estimates of the Association of the COVID-19 Pandemic With Hospital Consumer Assessment of Healthcare Providers and Systems (HCAHPS) Summary Scores by Quartiles of 2019 Staffing Level A plot of the departures of the HCAHPS top-box summary score from what would have been expected had 2018 to 2019 linear and quarterly trends continued, adjusting for patient mix and mode of survey administration, stratified by quartiles of 2019 staffing level. Estimated quarterly pandemic effects are shown as stratified by quartiles of 2019 staffing level. See Table 2.

Hospitals with better overall quality (as indicated by hospital overall star rating) before the pandemic showed consistently smaller declines in HCAHPS-SS during the pandemic, with effects for 5-star hospitals about 25% smaller than those for 1-star and 2-star hospitals in most quarters (Table 2; Figure 3). However, by Q3/2021, hospitals in all star rating groups had dropped a full pp, narrowing the difference so that by Q4/2021, the effect for 5-star hospitals was only 13% smaller than that for 1-star and 2-star hospitals (−3.2% for 5 stars vs −3.7% for 1 to 2 stars).

### Sensitivity Analyses

Results were similar when restricting to smaller, constant cohorts of hospitals (eFigures 2-4 in Supplement 1). Patient volume changes across the interval were limited except for a sharp decrease in Q2/2022 that was followed by a recovery in Q3/2022, which did not correspond to trends in the HCAHPS-SS (eFigure 5 in Supplement 1).

### Variation by Census Division

As shown in eTables 4A and B and eFigure 6 in Supplement 1, HCAHPS scores decreased in all US Census divisions. There was little regional variation.

## Discussion

In this cohort study of US hospitals, patient hospital experiences as measured by the HCAHPS survey were worse than expected based on prepandemic trends during the COVID-19 pandemic. This unprecedented decline in HCAHPS scores was seen in every region of the US, with little regional variation. Hospitals with higher staffing levels and better overall prepandemic quality were more resilient and slower to decline, but eventually they also declined.

By Q4/2021, a summary measure of patient experiences was 3.6 pp lower across all hospitals than would have been expected without the pandemic, a medium effect size by the guidelines by Quigley et al^21^ for patient experience measures. Staff responsiveness and cleanliness scores decreased by large amounts, possibly reflecting high absenteeism in the hospital workforce and delays in care associated with staff having to wear PPE.^9,22^ The decline of the overall rating and hospital recommendation measures lagged the decline in more specific HCAHPS measures, possibly reflecting initial forbearance or lower expectations in patients’ overall assessments at the onset of the pandemic. However, as the pandemic continued, the global ratings also declined to a similar extent as the more specific measures of patient experience. This pattern suggests that global ratings alone do not fully capture changes in specific patient experiences,^23^ nor do they identify the areas in need of improvement, especially in the face of unusual events, such as a pandemic. Deficits were largest for hospitals that during the prepandemic period were lower performing and had lower staffing levels. These hospitals may have been less resilient to reductions in staff availability during the COVID-19 pandemic and less able to adapt to unprecedented challenges in the health care environment. However, 2 years into the pandemic, the initially resilient hospitals’ HCAHPS scores had declined almost as much as those of the prepandemic lower-performing hospitals. The emergence of the highly contagious Omicron variant, ongoing staffing shortages, and cumulative effects of staff burnout may have compromised patient experiences of care in all hospitals.^24,25^

### Limitations

This study has several potential limitations. First, response rates were modest, and nonresponse bias may have affected the findings. However, research on Consumer Assessment of Healthcare Providers and Systems surveys has found little evidence of nonresponse bias after adjustment for patient mix.^18^ Response rates declined slightly throughout 2018 to 2021, which were consistent with prepandemic trends and trends for surveys in general.^26^ Second, the study used observational data; the analyses and conclusions were limited to the description of the departures of 2020 to 2021 HCAHPS scores from 2018 to 2019 trends. Unobserved factors may be responsible for the changes, as is the case for all observational data. Third, we used linear rather than logistic regression models to assess trends in top-box scores because linear models are easier to interpret and are almost identical when sample sizes are large and outcomes are predominantly between 20% and 80%.^18,27^ Fourth, the hospital overall star rating measure used to stratify hospitals included prepandemic hospital patient experience scores, with a 22% weight. However, there was a positive association between HCAHPS and the other components of the overall star rating. A modified version of the overall star ratings that removed the HCAHPS star ratings had a correlation of 0.985 with the hospital overall star rating (results not shown). As such, these results were not sensitive to the inclusion of HCAHPS in the prepandemic quality scores. Further, although one would expect some regression to the mean, which would be associated with slightly larger decreases for hospitals with higher prepandemic HCAHPS scores, such effects were accounted for in the regression models. Finally, prepandemic HCAHPS scores were based on potentially different, possibly less severe cases than post-2019 HCAHPS scores, which may have affected patient ratings. For example, in the Medicare Consumer Assessment of Healthcare Providers and Systems survey, patients with end-stage kidney disease and diabetes tend to give more positive ratings than otherwise similar patients.^28^ Nonetheless, self-rated health of HCAHPS patients was similar from 2018 to 2019 and 2020 to 2021, and scores were adjusted for self-rated health status and service lines. There were also strengths to this study. For example, the large volume and long tenure of HCAHPS data and their specific measures make it possible to understand which aspects of inpatient care were most affected by the pandemic, their course over time, and how resilience in the face of the COVID-19 pandemic varied regionally and by hospital characteristics.

## Conclusions

The results of this cohort study found that HCAHPS patient experience scores declined from 2020 to 2021. The most affected measures (staff responsiveness and cleanliness) showed large effect sizes, possibly reflecting high illness-associated hospital workforce absenteeism. Hospitals that were higher performing and higher staffed prepandemic were slower to decline, but by Q4/2021, scores for even these hospitals had declined.

## Notes

Supplement 1. **eMethods 1.** Details on the HCAHPS Sample Criterion **eMethods 2.** Statistical Tests **eMethods 3.** Details on Sensitivity Analysis **eTable 1.** Pre-Pandemic (Q1/2018-Q4/2019) and Post-2019 (Q1/2020-Q4/2021) Patient Characteristics **eTable 2. **Characteristics of the 3,381 Hospitals Included in the Primary Analysis **eTable 3. **Estimated Pandemic Effects (in top box percentage points) on Adjusted Individual HCAHPS Measures **eTable 4A. **Standard deviation across Census Divisions of the Estimated Pandemic Effects on HCAHPS-SS (in top-box points), by Quarter **eTable 4B. **Mean Q2/2020-Q4/2021 Estimated Pandemic Effects on HCAHPS-SS (in top-box points), by Census Division **eFigure 1. **Pre-Pandemic and Post-2019 National Mean Top-Box Scores 2018-2021 **eFigure 2. **Quarterly Estimates of the Pandemic Effect on HCAHPS-SS by Quarter: Sensitivity Test for Hospitals Included **eFigure 3A. **Quarterly Estimates of Pandemic Effect on HCAHPS-SS, by 2019 Hospital Overall Star Ratings, Standard Sample **eFigure 3B. **Quarterly Estimates of Pandemic Effect on HCAHPS-SS, by 2019 Hospital Overall Star Ratings, Restricted to Alt 1: 1,402 Hospitals in all 16 quarters Q118-Q421 **eFigure 3C. **Quarterly Estimates of Pandemic Effect on HCAHPS-SS, by 2019 Hospital Overall Star Ratings, Restricted to Alt 2: 1,299 Hospitals in All 16 Quarters Q118-Q421, Not Counting Voluntary Quarters Q120 and Q220 **eFigure 4A. **Quarterly Estimates of Pandemic Effects on HCAHPS Summary Scores by Quartiles of 2019 Staffing Level **eFigure 4B. **Quarterly Estimates of Pandemic Effect on HCAHPS Summary Scores by Quartiles of 2019 Staffing Level, Restricted to Alt 1: 1,402 Hospitals in all 16 quarters Q118-Q421 **eFigure 4C. **Quarterly Estimates of Pandemic Effect on HCAHPS Summary Scores by Quartiles of 2019 Staffing Level, Restricted to Alt 2: 1,299 Hospitals in All 16 Quarters Q118-Q421, Not Counting Voluntary Quarters Q120 and Q220 **eFigure 5. **Average Quarterly HCAHPS Hospital Eligible Discharges per Reporting Hospital, Q118-Q421 **eFigure 6. **Quarterly Estimates of Pandemic Effect on HCAHPS-SS by Census Division

Supplement 2. Data sharing statement

## References

[1] US Centers for Medicare & Medicaid Services. HCAHPS: patients’ perspectives of care survey. Accessed November 9, 2022. https://www.cms.gov/Medicare/Quality-Initiatives-Patient-Assessment-Instruments/HospitalQualityInits/HospitalHCAHPS

[2] Shanafelt T, Ripp J, Trockel M. Understanding and addressing sources of anxiety among health care professionals during the COVID-19 pandemic. JAMA. 2020;323(21):2133-2134. doi:10.1001/jama.2020.5893 32259193

[3] Pun BT, Badenes R, Heras La Calle G, ; COVID-19 Intensive Care International Study Group. Prevalence and risk factors for delirium in critically ill patients with COVID-19 (COVID-D): a multicentre cohort study. Lancet Respir Med. 2021;9(3):239-250. doi:10.1016/S2213-2600(20)30552-X 33428871 PMC7832119

[4] Sexton JB, Adair KC, Proulx J, . Emotional exhaustion among US health care workers before and during the COVID-19 pandemic, 2019-2021. JAMA Netw Open. 2022;5(9):e2232748. doi:10.1001/jamanetworkopen.2022.32748 36129705 PMC9494188

[5] Harris GH, Rak KJ, Kahn JM, . US hospital capacity managers’ experiences and concerns regarding preparedness for seasonal influenza and influenza-like illness. JAMA Netw Open. 2021;4(3):e212382. doi:10.1001/jamanetworkopen.2021.2382 33739431 PMC7980097

[6] Baker MA, Sands KE, Huang SS, ; CDC Prevention Epicenters Program. The impact of coronavirus disease 2019 (COVID-19) on healthcare-associated infections. Clin Infect Dis. 2022;74(10):1748-1754. doi:10.1093/cid/ciab688 34370014 PMC8385925

[7] Sharma A, Pillai DR, Lu M, . Impact of isolation precautions on quality of life: a meta-analysis. J Hosp Infect. 2020;105(1):35-42. doi:10.1016/j.jhin.2020.02.004 32059996

[8] Nair R, Perencevich EN, Goto M, . Patient care experience with utilization of isolation precautions: systematic literature review and meta-analysis. Clin Microbiol Infect. 2020;26(6):684-695. doi:10.1016/j.cmi.2020.01.022 32006691 PMC7253340

[9] Drapeaux A, Jenson JA, Fustino N. The impact of COVID-19 on patient experience within a Midwest hospital system: a case study. J Patient Exp. 2021;8:23743735211065298. doi:10.1177/23743735211065298 34901416 PMC8664302

[10] von Elm E, Altman DG, Egger M, Pocock SJ, Gøtzsche PC, Vandenbroucke JP; STROBE Initiative. The Strengthening the Reporting of Observational Studies in Epidemiology (STROBE) statement: guidelines for reporting observational studies. Lancet. 2007;370(9596):1453-1457. doi:10.1016/S0140-6736(07)61602-X 18064739

[11] US Centers for Medicare & Medicaid Services. Find & compare providers near you. Accessed December 2, 2022. https://www.medicare.gov/care-compare/

[12] US Centers for Medicare & Medicaid Services. HCAHPS patient-level correlations. Accessed June 16, 2023. https://hcahpsonline.org/globalassets/hcahps/summary-analyses/correlations/2023-04-correlations.pdf

[13] Elliott MN, Kanouse DE, Edwards CA, Hilborne LH. Components of care vary in importance for overall patient-reported experience by type of hospitalization. Med Care. 2009;47(8):842-849. doi:10.1097/MLR.0b013e318197b22a 19584764

[14] Jha AK, Orav EJ, Zheng J, Epstein AM. Patients’ perception of hospital care in the United States. N Engl J Med. 2008;359(18):1921-1931. doi:10.1056/NEJMsa0804116 18971493

[15] Tefera L, Lehrman WG, Conway P. Measurement of the patient experience: clarifying facts, myths, and approaches. JAMA. 2016;315(20):2167-2168. doi:10.1001/jama.2016.1652 26967744

[16] Zhu J, Dy SM, Wenzel J, Wu AW. Association of magnet status and nurse staffing with improvements in patient experience with hospital care, 2008–2015. Med Care. 2018;56(2):111-120. doi:10.1097/MLR.0000000000000854 29271818

[17] Cefalu M, Elliott MN, Hays RD. Adjustment of patient experience surveys for how people respond. Med Care. 2021;59(3):202-205. doi:10.1097/MLR.0000000000001489 33427795 PMC7878315

[18] Elliott MN, Zaslavsky AM, Goldstein E, . Effects of survey mode, patient mix, and nonresponse on CAHPS hospital survey scores. Health Serv Res. 2009;44(2 pt 1):501-518. doi:10.1111/j.1475-6773.2008.00914.x 19317857 PMC2677051

[19] US Centers for Medicare & Medicaid Services. CMS announces relief for clinicians, providers, hospitals, and facilities. Accessed February 7, 2023. https://www.cms.gov/newsroom/press-releases/cms-announces-relief-clinicians-providers-hospitals-and-facilities-participating-quality-reporting

[20] US Centers for Medicare & Medicaid Services. Summary of HCAHPS survey results tables. Accessed February 7, 2023. https://hcahpsonline.org/en/summary-analyses/

[21] Quigley DD, Elliott MN, Setodji CM, Hays RD. Quantifying magnitude of group-level differences in patient experiences with health care. Health Serv Res. 2018;53(suppl)(suppl 1):3027-3051. doi:10.1111/1475-6773.12828 29435975 PMC6056572

[22] Butler CR, Wong SPY, Wightman AG, O’Hare AM. US clinicians’ experiences and perspectives on resource limitation and patient care during the COVID-19 pandemic. JAMA Netw Open. 2020;3(11):e2027315. doi:10.1001/jamanetworkopen.2020.27315 33156349 PMC7648254

[23] Elliott MN, Haviland AM, Kanouse DE, Hambarsoomian K, Hays RD. Adjusting for subgroup differences in extreme response tendency in ratings of health care: impact on disparity estimates. Health Serv Res. 2009;44(2 pt 1):542-561. doi:10.1111/j.1475-6773.2008.00922.x 19040424 PMC2677053

[24] Iuliano AD, Brunkard JM, Boehmer TK, . Trends in disease severity and health care utilization during the early Omicron variant period compared with previous SARS-CoV-2 high transmission periods—United States, December 2020–January 2022. MMWR Morb Mortal Wkly Rep. 2022;71(4):146-152. doi:10.15585/mmwr.mm7104e4 35085225 PMC9351529

[25] Danza P, Koo TH, Haddix M, . SARS-CoV-2 infection and hospitalization among adults aged≥ 18 years, by vaccination status, before and during SARS-CoV-2 B. 1.1. 529 (Omicron) variant predominance—Los Angeles County, California, November 7, 2021–January 8, 2022. MMWR Morb Mortal Wkly Rep. 2022;71(5):177-181. doi:10.15585/mmwr.mm7105e1 35113851 PMC8812833

[26] Brick JM, Williams D. Explaining rising nonresponse rates in cross-sectional surveys. Ann Am Acad Pol Soc Sci. 2013;645(1):36-59. doi:10.1177/0002716212456834

[27] Hellevik O. Linear versus logistic regression when the dependent variable is a dichotomy. Qual Quant. 2009;43(1):59-74. doi:10.1007/s11135-007-9077-3

[28] Paddison CA, Elliott MN, Haviland AM, . Experiences of care among Medicare beneficiaries with ESRD: Medicare Consumer Assessment of Healthcare Providers and Systems (CAHPS) survey results. Am J Kidney Dis. 2013;61(3):440-449. doi:10.1053/j.ajkd.2012.10.009 23177730

