# Supplementary material for: Changes in Patient Experiences of Hospital Care During the COVID-19 Pandemic
Source: JAMA Health Forum. 2023 Aug 25;4(8):e232766. doi: 10.1001/jamahealthforum.2023.2766 (PMC10457712; doi:10.1001/jamahealthforum.2023.2766)
Supplement: Supplement 1. — eMethods 1. Details on the HCAHPS Sample Criterion eMethods 2. Statistical Tests eMethods 3. Details on Sensitivity Analysis eTable 1. Pre-Pandemic (Q1/2018-Q4/2019) and Post-2019 (Q1/2020-Q4/2021) Patient Characteristics eTable 2. Characteristics of the 3,381 Hospitals Included in the Primary Analysis eTable 3. Estimated Pandemic Effects (in top box percentage points) on Adjusted Individual HCAHPS Measures eTable 4A. Standard deviation across Census Divisions of the Estimated Pandemic Effects on HCAHPS-SS (in top-box points), by Quarter eTable 4B. Mean Q2/2020-Q4/2021 Estimated Pandemic Effects on HCAHPS-SS (in top-box points), by Census Division eFigure 1. Pre-Pandemic and Post-2019 National Mean Top-Box Scores 2018-2021 eFigure 2. Quarterly Estimates of the Pandemic Effect on HCAHPS-SS by Quarter: Sensitivity Test for Hospitals Included eFigure 3A. Quarterly Estimates of Pandemic Effect on HCAHPS-SS, by 2019 Hospital Overall Star Ratings, Standard Sample eFigure 3B. Quarterly Estimates of Pandemic Effect on HCAHPS-SS, by 2019 Hospital Overall Star Ratings, Restricted to Alt 1: 1,402 Hospitals in all 16 quarters Q118-Q421 eFigure 3C. Quarterly Estimates of Pandemic Effect on HCAHPS-SS, by 2019 Hospital Overall Star Ratings, Restricted to Alt 2: 1,299 Hospitals in All 16 Quarters Q118-Q421, Not Counting Voluntary Quarters Q120 and Q220 eFigure 4A. Quarterly Estimates of Pandemic Effects on HCAHPS Summary Scores by Quartiles of 2019 Staffing Level eFigure 4B. Quarterly Estimates of Pandemic Effect on HCAHPS Summary Scores by Quartiles of 2019 Staffing Level, Restricted to Alt 1: 1,402 Hospitals in all 16 quarters Q118-Q421 eFigure 4C. Quarterly Estimates of Pandemic Effect on HCAHPS Summary Scores by Quartiles of 2019 Staffing Level, Restricted to Alt 2: 1,299 Hospitals in All 16 Quarters Q118-Q421, Not Counting Voluntary Quarters Q120 and Q220 eFigure 5. Average Quarterly HCAHPS Hospital Eligible Discharges per Reporting Hospital, Q118-Q421 eFigure 6. Quarterly Est [file jamahealthforum-e232766-s001.pdf]

Supplemental Online Content

Elliott MN, Beckett MK, Cohea CW, et al. Changes in patient experiences of hospital care during the COVID-19 pandemic. *JAMA Health Forum*. 2023;4(8):e232766. doi:10.1001/jamahealthforum.2023.2766

eMethods 1. Details on the HCAHPS Sample Criterion
eMethods 2. Statistical Tests
eMethods 3. Details on Sensitivity Analysis
eTable 1. Pre-Pandemic (Q1/2018-Q4/2019) and Post-2019 (Q1/2020-Q4/2021) Patient Characteristics
eTable 2. Characteristics of the 3,381 Hospitals Included in the Primary Analysis
eTable 3. Estimated Pandemic Effects (in top box percentage points) on Adjusted Individual HCAHPS Measures
eTable 4A. Standard deviation across Census Divisions of the Estimated Pandemic Effects on HCAHPS-SS (in top-box points), by Quarter
eTable 4B. Mean Q2/2020-Q4/2021 Estimated Pandemic Effects on HCAHPS-SS (in top-box points), by Census Division
eFigure 1. Pre-Pandemic and Post-2019 National Mean Top-Box Scores 2018-2021
eFigure 2. Quarterly Estimates of the Pandemic Effect on HCAHPS-SS by Quarter: Sensitivity Test for Hospitals Included
eFigure 3A. Quarterly Estimates of Pandemic Effect on HCAHPS-SS, by 2019 Hospital Overall Star Ratings, Standard Sample
eFigure 3B. Quarterly Estimates of Pandemic Effect on HCAHPS-SS, by 2019 Hospital Overall Star Ratings, Restricted to Alt 1: 1,402 Hospitals in all 16 quarters Q118-Q421
eFigure 3C. Quarterly Estimates of Pandemic Effect on HCAHPS-SS, by 2019 Hospital Overall Star Ratings, Restricted to Alt 2: 1,299 Hospitals in All 16 Quarters Q118-Q421, Not Counting Voluntary Quarters Q120 and Q220
eFigure 4A. Quarterly Estimates of Pandemic Effects on HCAHPS Summary Scores by Quartiles of 2019 Staffing Level
eFigure 4B. Quarterly Estimates of Pandemic Effect on HCAHPS Summary Scores by Quartiles of 2019 Staffing Level, Restricted to Alt 1: 1,402 Hospitals in all 16 quarters Q118-Q421
eFigure 4C. Quarterly Estimates of Pandemic Effect on HCAHPS Summary Scores by Quartiles of 2019 Staffing Level, Restricted to Alt 2: 1,299 Hospitals in All 16 Quarters Q118-Q421, Not Counting Voluntary Quarters Q120 and Q220
eFigure 5. Average Quarterly HCAHPS Hospital Eligible Discharges per Reporting Hospital, Q118-Q421
eFigure 6. Quarterly Estimates of Pandemic Effect on HCAHPS-SS by Census Division

© 2023 Elliott MN et al. *JAMA Health Forum.*

This supplemental material has been provided by the authors to give readers additional information about their work.

© 2023 Elliott MN et al. *JAMA Health Forum.*

eMethods 1: Details on the HCAHPS Sample Criterion

The goal of this study was to assess post-2019 departures from expected HCAHPS scores based on pre-pandemic trends. For our primary analyses, we answer this question using hospitals with at least 25 completed HCAHPS surveys in a given quarter, which corresponds to the standard criterion of 100+ completed surveys annually for reliable HCAHPS measurement at the hospital level.[29]

© 2023 Elliott MN et al. *JAMA Health Forum.*

eMethods 2: Statistical Tests

The goal of our primary analysis was to estimate departures from the HCAHPS Summary Score (SS) that would have been expected for hospitals based on pre-pandemic HCAHPS-SS trends. To derive these estimated departures from linear and seasonal pre-pandemic trends, we estimated eight linear mixed-effect regression models. Each model included all patient-level data from all hospital-quarter combinations with at least 25 completed surveys from a set of nine quarters. All eight modes contained data for the eight quarters Q1/2018-Q4/2019 plus data from one post-2019 quarter (Q1/2020 through Q4/2021, one per model). We used the following mixed-effect linear regression model with a random intercept for hospitals to calculate our estimates for each HCAHPS outcome and to compare post-2019 departures from expected HCAHPS scores overall and for different types of hospitals:

$$Y_{ij} = \beta_0 + \beta_z QuarterssinceQ12018 + \beta_3 Quarter1 + \beta_4 Quarter2 + \beta_5 Quarter3 + \beta_4 Voluntary\ Reporter + \beta_5 post2019Indicator + patientmix\ adjusters + + \alpha_i + \varepsilon_{ij}.$$

Y_{ij} denotes a patient-level HCAHPS score for hospital *i* at quarter *j*, after pre-adjustment for hospital-level survey mode derived from data from a randomized mode experiment [30] [telephone, mixed mode (mail with telephone follow-up of mail nonrespondents), active interactive voice response, with mail only as the referent]. *QuarterssinceQ12018* is a count of the number of quarters since Q1/2018, to account for pre-pandemic linear trends; to control for seasonality within calendar years, *Quarter1* is set to 1 if first quarter of the year, *Quarter2* is set to 1 if second quarter of the year, and *Quarter3* is set to 1 if third quarter of the year. *Voluntary Reporter* is set to 1 if the hospital reported in Q1/2020 or Q2/2020. *Post2019Indicator* is set to 1 for the one included quarter from Q1/2020 thru Q4/2021 in a given model; its coefficient estimates the departure from expected HCAHPS score; *patient-mix adjusters* are parameterized as described in www.hcahpsonline.org to account for factors not under the control of hospitals that affect how patients use CAHPS survey response scales. These are education, age, sex by service line (male medical, male surgical, female surgical, female maternity, with female medical as the referent), interaction between surgical line and age, interaction between maternity line and age, self-rated health, self-rated mental health, preferred language (Spanish, Chinese or Russian/Vietnamese/Portuguese/Other, with English as the referent), and response percentile (which accounts for time from discharge to response); α_i represents the random intercept for each hospital i to account for within-hospital correlations and to allow hospital to differ in mean performance; ε represents the error term.

© 2023 Elliott MN et al. *JAMA Health Forum.*

eMethods 3: Details on Sensitivity Analysis

Reporting was voluntary and limited in Q1/2020 and Q2/2020. In sensitivity analyses, we estimated difference from expected HCAHPS-SS based on pre-pandemic trends restricting to (1) Alt 1: the 1299 hospitals with at least 25 completed surveys in every quarter Q1/2018-Q4/2021 (voluntary reporters with sufficient sample size) and (2) Alt 2: the 1402 hospitals with at least 25 completed surveys in all quarters Q1/2018-Q4/2019 plus Q3/2020-Q4/2021 (those with sufficient sample size, ignoring voluntary reporting).

© 2023 Elliott MN et al. *JAMA Health Forum.*

eTable 1: Pre-Pandemic (Q1/2018-Q4/2019) and Post-2019 (Q1/2020-Q4/2021) Patient Characteristics

	2018-2019 (n=5,298,431)	2020-2021 (n=3,900,887)	2018-2019 to 2020-2021 Change
Language spoken at home			
English	92.5%	92.2%	-0.2%
Spanish	5.3%	5.5%	0.2%
Chinese	0.4%	0.4%	0.0%
Other	1.8%	1.9%	0.0%
Service line by sex			
Maternity	10.6%	10.5%	-0.1%
Medical, female	27.4%	28.3%	0.9%
Surgical, female	19.3%	17.2%	-2.1%
Medical, male	24.0%	26.5%	2.5%
Surgical, male	18.7%	17.5%	-1.3%
Age			
Age 18-24	2.4%	2.1%	-0.4%
Age 25-34	8.7%	8.5%	-0.2%
Age 35-44	5.9%	6.1%	0.2%
Age 45-54	7.7%	7.3%	-0.4%
Age 55-64	18.1%	17.2%	-0.9%
Age 65-74	27.3%	27.5%	0.2%
Age 75-84	21.2%	22.3%	1.1%
Age 85+	8.6%	8.9%	0.4%
Self-Rated Overall Health			
Excellent	13.3%	12.8%	-0.5%
Very Good	27.2%	26.5%	-0.7%
Good	33.9%	34.8%	1.0%
Fair	19.6%	20.3%	0.7%
Poor	6.0%	5.6%	-0.4%
Self-Rated Mental Health			
Excellent	29.6%	27.4%	-2.3%
Very Good	34.0%	34.3%	0.3%
Good	25.8%	27.1%	1.2%
Fair	9.0%	9.6%	0.6%
Poor	1.6%	1.6%	0.0%
Education			
No High School	4.3%	4.0%	-0.3%
Some High School	7.4%	6.8%	-0.6%
High School Degree	30.4%	30.0%	-0.4%
Some College	29.3%	29.5%	0.2%
4-Year College Graduate	13.6%	14.3%	0.7%

© 2023 Elliott MN et al. *JAMA Health Forum.*

Graduate Degree	14.9%	15.4%	0.5%

Note: There were only small differences between patients in 2020-21 and 2018-19 in terms of preferred language, sex and service line, age, general health, mental health, and education. All differences were smaller than 3 percentage points, with most differences smaller than 1 percentage point.

© 2023 Elliott MN et al. *JAMA Health Forum.*

eTable 2: Characteristics of the 3,381 Hospitals Included in the Primary Analysis

	%	n
Census Division		
East North Central	16.9%	571
East South Central	7.7%	260
Mid Atlantic	10.5%	355
Mountain	8.1%	274
New England	4.7%	159
Pacific	13.0%	440
South Atlantic	16.5%	558
West North Central	9.8%	331
West South Central	12.8%	433
Location		
Rural	30.0%	1014
Urban	71.0%	2367
Hospital Type		
Inpatient Prospective Payment System	87.5%	2958
Critical Access Hospital	12.5%	423
Total		3381

In 2019, 99.1% of all HCAHPS-eligible patients were from hospitals with 25 or more completed surveys in a given quarter. Thus, only 0.9% of all HCAHPS eligible-patients are served by hospitals that were excluded due to inadequate hospital-level reliability.

© 2023 Elliott MN et al. *JAMA Health Forum.*

eTable 3: Estimated Pandemic Effects (in top box percentage points) on Adjusted Individual HCAHPS Measures[a]

	Q1/2020 (N=5,861,470 patients; n=1648 hospitals)			Q2/2020 (N=5,807,876 patients; n=1737 hospitals)			Q3/2020 (N=6,118,270 patients; n=3224 hospitals)			Q4/2020 (N=6,104,234 patients; n=3295 hospitals)		
	Est	SE	P	Est	SE	p	Est	SE	p	Est	SE	p
Communication With Nurses	0.08%	0.07%	.26	-1.19%	0.08%	<.001	-1.96%	0.06%	<.001	-1.83%	0.06%	<.001
Communication With Nurses	-0.02%	0.07%	.76	-1.12%	0.08%	<.001	-1.67%	0.06%	<.001	-1.53%	0.06%	<.001
Staff Responsiveness	-0.73%	0.10%	<.001	-1.69%	0.11%	<.001	-2.98%	0.09%	<.001	-3.58%	0.09%	<.001
Communication About Medicine	-0.83%	0.11%	<.001	-2.94%	0.12%	<.001	-3.18%	0.10%	<.001	-2.72%	0.10%	<.001
Cleanliness of Environment	-0.56%	0.10%	<.001	-0.96%	0.11%	<.001	-2.82%	0.09%	<.001	-3.27%	0.09%	<.001
Quietness of Environment	1.00%	0.11%	<.001	1.35%	0.11%	<.001	-0.05%	0.10%	.59	0.21%	0.10%	.03
Discharge Information	0.22%	0.06%	.003	-0.80%	0.06%	<.001	-0.78%	0.05%	<.001	-0.84%	0.05%	<.001
Care Transition	-0.10%	0.10%	.29	-2.49%	0.10%	<.001	-2.81%	0.08%	<.001	-2.58%	0.08%	<.001
Hospital Recommendation	0.60%	0.10%	<.001	-0.33%	0.10%	.001	-1.41%	0.08%	<.001	-0.69%	0.09%	<.001
Overall Rating	0.77%	0.10%	<.001	-0.30%	0.10%	.004	-1.76%	0.09%	<.001	-1.10%	0.09%	<.001

	Q1/2021 (N=6,099,727 patients; n=3219 hospitals)			Q2/2021 (N=6,109,713 patients; n=3181 hospitals)			Q3/2021 (N=6,084,396 patients; n=3232 hospitals)			Q4/2021 (N=5,990,289 patients; n=3137 hospitals)		
	Est	SE	p	Est	SE	p	Est	SE	p	Est	SE	p
Communication With Nurses	-2.10%	0.08%	<.001	-2.31%	0.08%	<.001	-3.18%	0.08%	<.001	-3.44%	0.09%	<.001
Communication With Nurses	-1.77%	0.08%	<.001	-1.97%	0.08%	<.001	-2.74%	0.08%	<.001	-2.86%	0.09%	<.001
Staff Responsiveness	-3.14%	0.12%	<.001	-3.38%	0.12%	<.001	-5.27%	0.12%	<.001	-5.60%	0.12%	<.001

© 2023 Elliott MN et al. *JAMA Health Forum.*

Communication About Medicine	-2.69%	0.13%	<.001	-2.97%	0.13%	<.001	-4.29%	0.13%	<.001	-4.24%	0.13%	<.001
Cleanliness of Environment	-2.58%	0.12%	<.001	-3.08%	0.12%	<.001	-4.64%	0.12%	<.001	-4.94%	0.12%	<.001
Quietness of Environment	0.17%	0.13%	.17	-1.31%	0.13%	<.001	-1.75%	0.13%	<.001	-1.75%	0.13%	<.001
Discharge Information	-0.76%	0.07%	<.001	-0.98%	0.07%	<.001	-1.35%	0.07%	<.001	-1.65%	0.07%	<.001
Care Transition	-2.80%	0.11%	<.001	-2.82%	0.11%	<.001	-3.79%	0.11%	<.001	-3.94%	0.11%	<.001
Hospital Recommendation	-0.89%	0.11%	<.001	-2.10%	0.11%	<.001	-2.89%	0.11%	<.001	-3.28%	0.11%	<.001
Overall Rating	-1.25%	0.11%	<.001	-2.35%	0.11%	<.001	-3.46%	0.11%	<.001	-3.87%	0.11%	<.001

[a] Each model includes Q1/2018-Q4/2019 discharges, plus one post-2019 quarter (each model has nine quarters). Each model is limited to hospitals with at least 25 completed surveys in its post-2019 quarter and includes data from all quarters for which these hospitals have at least 25 completed surveys Models also include hospital random effects.

© 2023 Elliott MN et al. *JAMA Health Forum.*

eTable 4A: Standard deviation across Census Divisions of the Estimated Pandemic Effects on HCAHPS-SS (in top-box points), by Quarter

Quarter	Standard deviation of effect across Census Divisions
Q2/2020	0.34%
Q3/2020	0.64%
Q4/2020	0.22%
Q1/2021	0.47%
Q2/2021	0.33%
Q3/2021	0.44%
Q4/2021	0.53%

eTable 4B: Mean Q2/2020-Q4/2021 Estimated Pandemic Effects on HCAHPS-SS (in top-box points), by Census Division

Census Division	Estimated Pandemic Effect for HCAHPS-SS
East North Central	-2.27%
East South Central	-2.16%
Mid Atlantic	-2.04%
Mountain	-2.24%
New England	-2.30%
Pacific	-2.29%
South Atlantic	-2.84%
West North Central	-1.91%
West South Central	-2.39%

Note: Regional variation in estimated HCAHPS effects was less than 1 point, the standard threshold for a small difference in HCAHPS scores. The standard deviation of the estimated pandemic effect across Census Divisions on HCAHPS-SS was between 0.20 and 0.66 top-box points Q2/2020-Q4/2021, with the most variability in Q3/2020 and least variability in Q4/2020 (eTable 4A, eFigure 6). Of the 9 Census Divisions, 6 had average estimated pandemic effects Q2/2020-Q4/2021 that were within 5% of the overall average estimated pandemic effect (eTable 4B, eFigure 6). One division (the South Atlantic) had an estimated pandemic effect that was 25% larger than the average estimated pandemic effect; the estimated effects in the West North Central and Mid Atlantic divisions were 16% and 10% smaller than average, respectively. The largest estimated effect differed from the smallest estimated effect by less than one point. States in each Census Division: East North Central: IL, IN, MI, OH, WI; East South Central: AL, KY, MS, TN; Mid-Atlantic: NJ, NY, PA; Mountain: AZ, CO, ID, MT, NM, NV, UT, WY ; New England: CT, MA, ME, NH, RI, VT; Pacific: CA, OR, WA, HI, AK; South Atlantic: DC, DE, FL, GA, MD, NC, SC, VA, WV; West North Central: IA, KS, MN, MO, NE, ND, SD; West South Central: AR, LA, OK, TX.

© 2023 Elliott MN et al. *JAMA Health Forum.*

eFigure 1: Pre-Pandemic and post-2019 National Mean Top-Box Scores 2018-2021

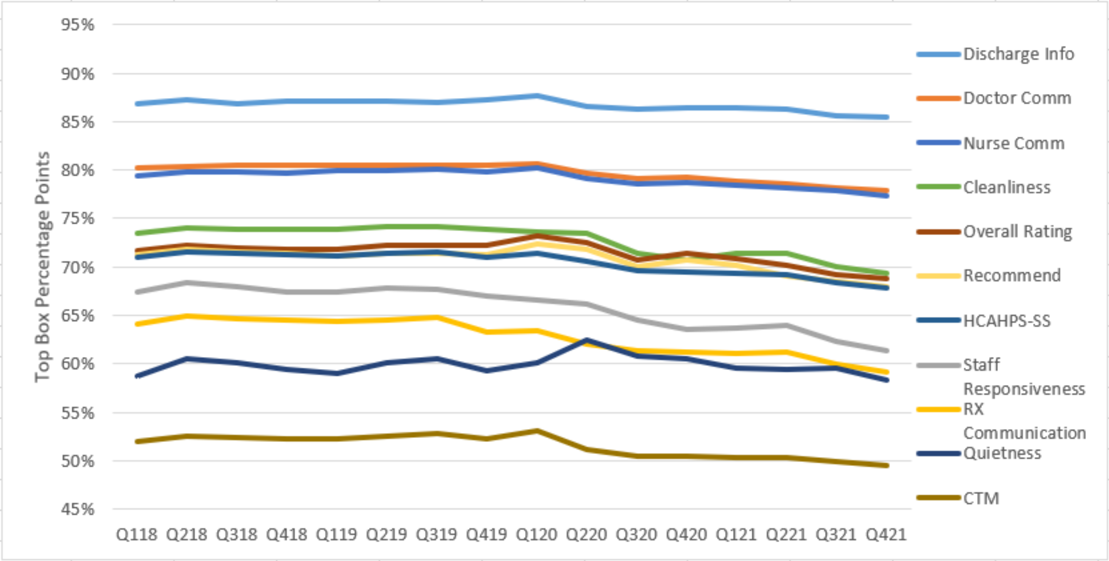

Note: The pre-pandemic trend is one of slightly increasing HCAHPS scores with some seasonality (higher scores during Q2,Q3 [April-September] than other times of year). Post-2019 there are absolute declines in scores that are similar to but slightly smaller than the departures from pre-pandemic trends used as the primary analysis.

© 2023 Elliott MN et al. *JAMA Health Forum.*

eFigure 2: Quarterly Estimates of the Pandemic Effect on HCAHPS-SS by Quarter: Sensitivity Test for Hospitals Included

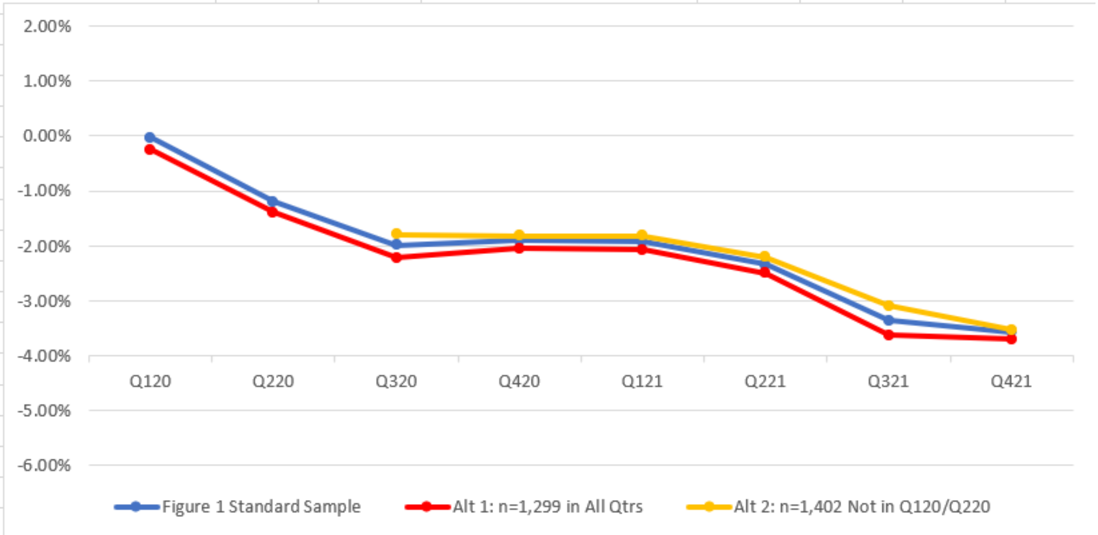

Note: The patterns in the two constant cohorts (Alt 1 and Alt 2) are very similar to one another and to the pattern in the standard sample used for the primary analysis in Figure 1.
Alt 1: the 1299 hospitals with at least 25 completed surveys in every quarter Q1/2018-Q4/2021 (voluntary reporters with sufficient sample size)
Alt 2: the 1402 hospitals with at least 25 completed surveys in all quarters Q1/2018-Q4/2019 plus Q3/2020-Q4/2021 (those with sufficient sample size, ignoring voluntary reporting).

© 2023 Elliott MN et al. *JAMA Health Forum.*

eFigure 3A: Quarterly Estimates of Pandemic Effect on HCAHPS-SS, by 2019 Hospital Overall Star Ratings, Standard Sample (Same as Figure 2)

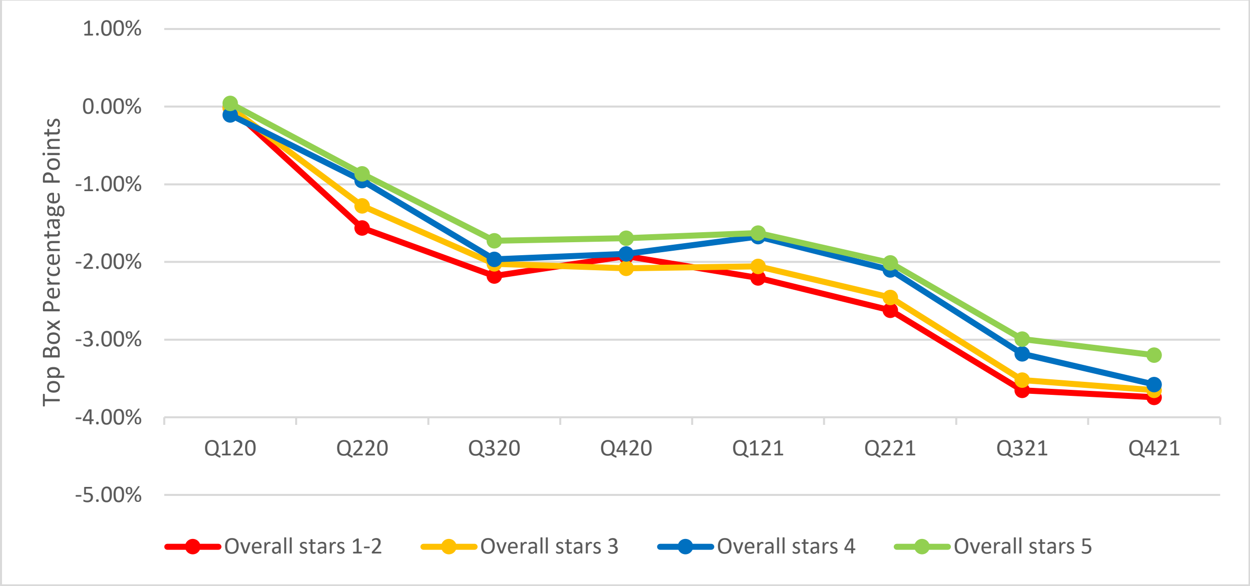

eFigure 3B: Quarterly Estimates of Pandemic Effect on HCAHPS-SS, by 2019 Hospital Overall Star Ratings, Restricted to Alt 1: 1,402 Hospitals in all 16 quarters Q118-Q421

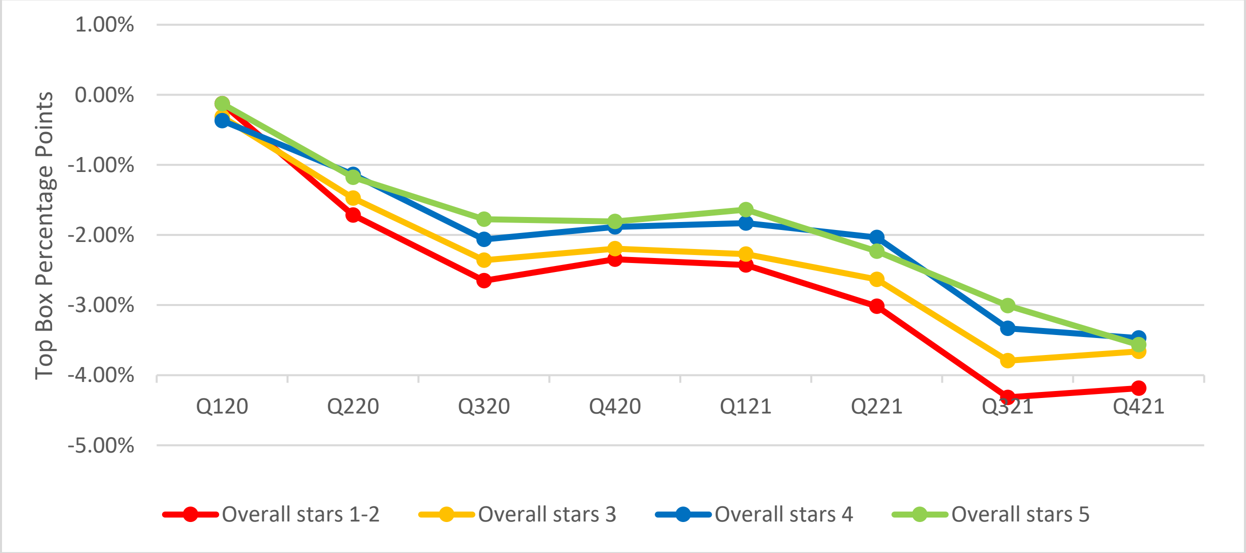

eFigure 3C: Quarterly Estimates of Pandemic Effect on HCAHPS-SS, by 2019 Hospital Overall Star Ratings, Restricted to Alt 2: 1,299 Hospitals in All 16 Quarters Q118-Q421, Not Counting Voluntary Quarters Q120 and Q220

© 2023 Elliott MN et al. *JAMA Health Forum.*

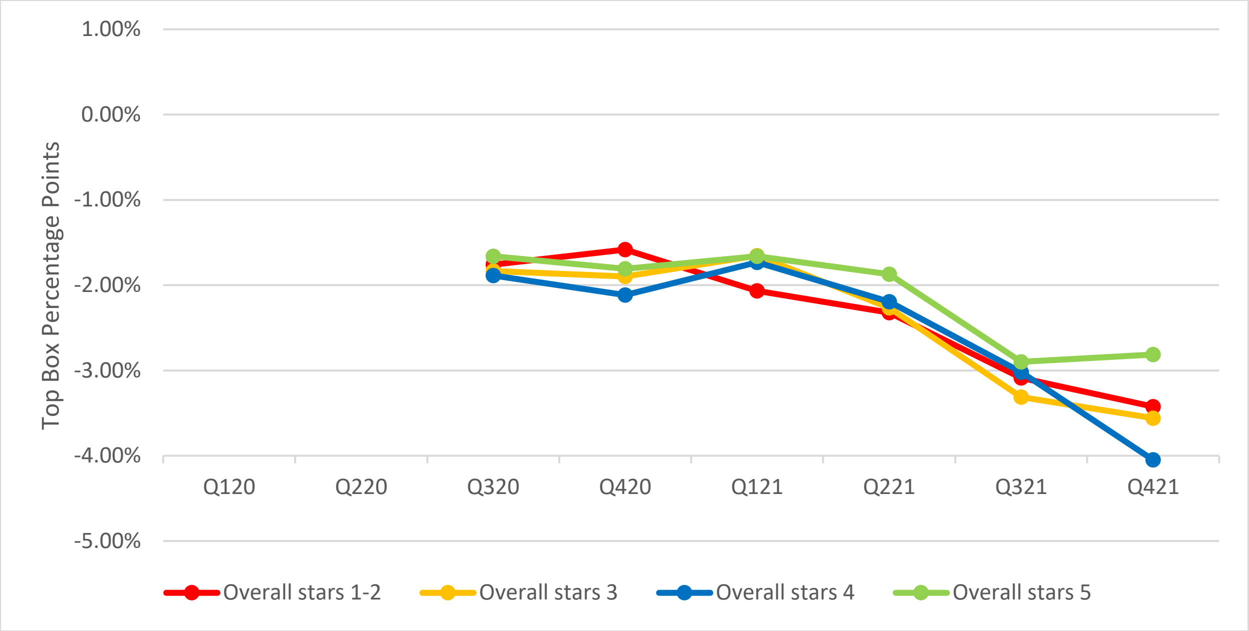

Note: Hospitals with higher 2019 Overall Star Ratings show smaller estimated pandemic effects on HCAHPS-SS when using the primary sample (as shown in Figure 2 and reproduced for easy comparison here as eFigure 3A) or either of the two constant cohorts (Alt 1 and Alt 2; eFigures 3A and 3B). Patterns do not appear to reflect differences between hospitals that voluntarily reported through the pandemic and other hospitals, nor do they appear to be a function of the entry and exit of hospitals over the study period.

Alt 1: the 1299 hospitals with at least 25 completed surveys in every quarter Q1/2018-Q4/2021 (voluntary reporters with sufficient sample size)
Alt 2: the 1402 hospitals with at least 25 completed surveys in all quarters Q1/2018-Q4/2019 plus Q3/2020-Q4/2021 (those with sufficient sample size, ignoring voluntary reporting).

© 2023 Elliott MN et al. *JAMA Health Forum.*

eFigure 4A: Quarterly Estimates of Pandemic Effects on HCAHPS Summary Scores by Quartiles of 2019 Staffing Level (Same as Figure 3)

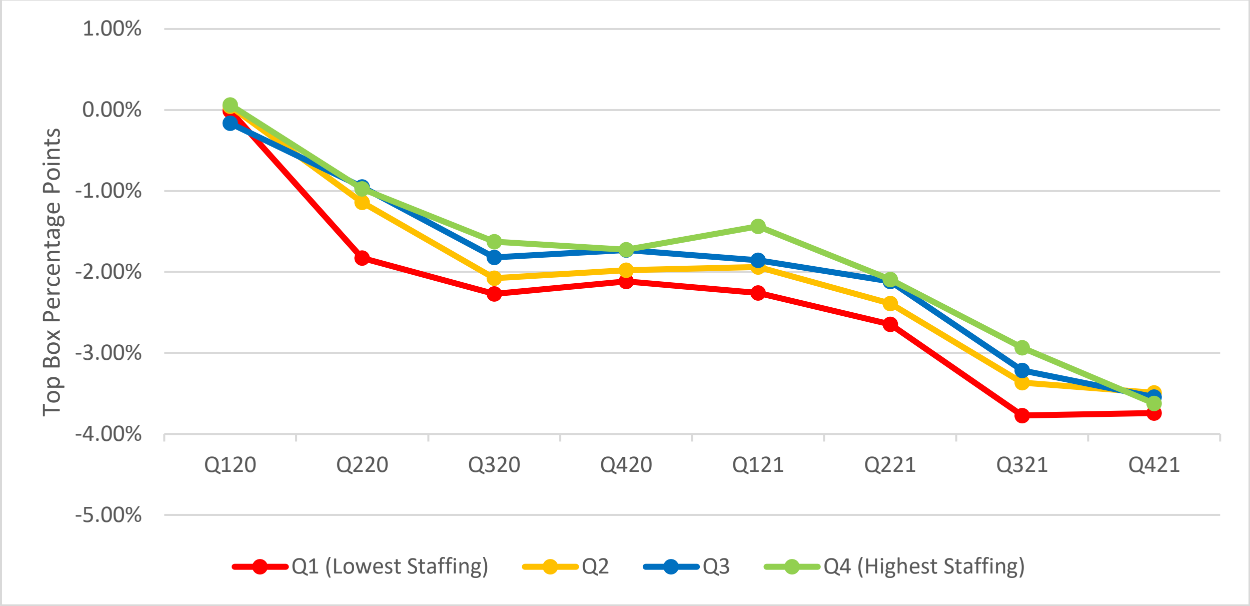

eFigure 4B: Quarterly Estimates of Pandemic Effect on HCAHPS Summary Scores by Quartiles of 2019 Staffing Level, Restricted to Alt 1: 1,402 Hospitals in all 16 quarters Q118-Q421

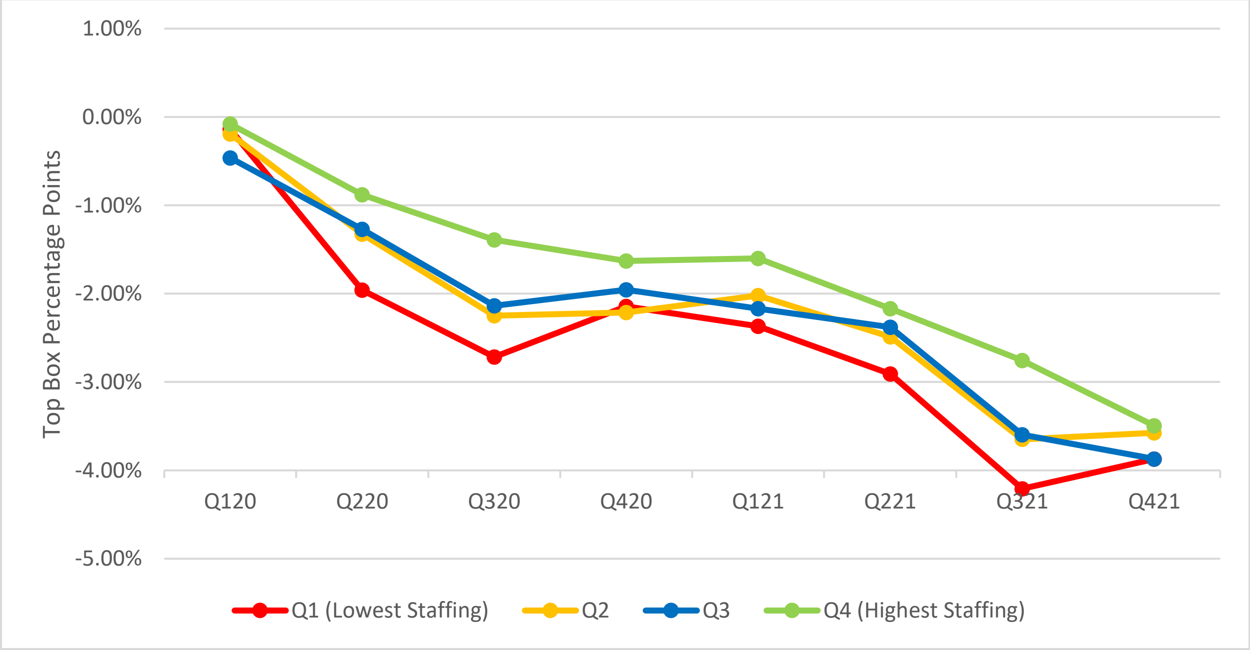

eFigure 4C: Quarterly Estimates of Pandemic Effect on HCAHPS Summary Scores by Quartiles of 2019 Staffing Level, Restricted to Alt 2: 1,299 Hospitals in All 16 Quarters Q118-Q421, Not Counting Voluntary Quarters Q120 and Q220

© 2023 Elliott MN et al. *JAMA Health Forum.*

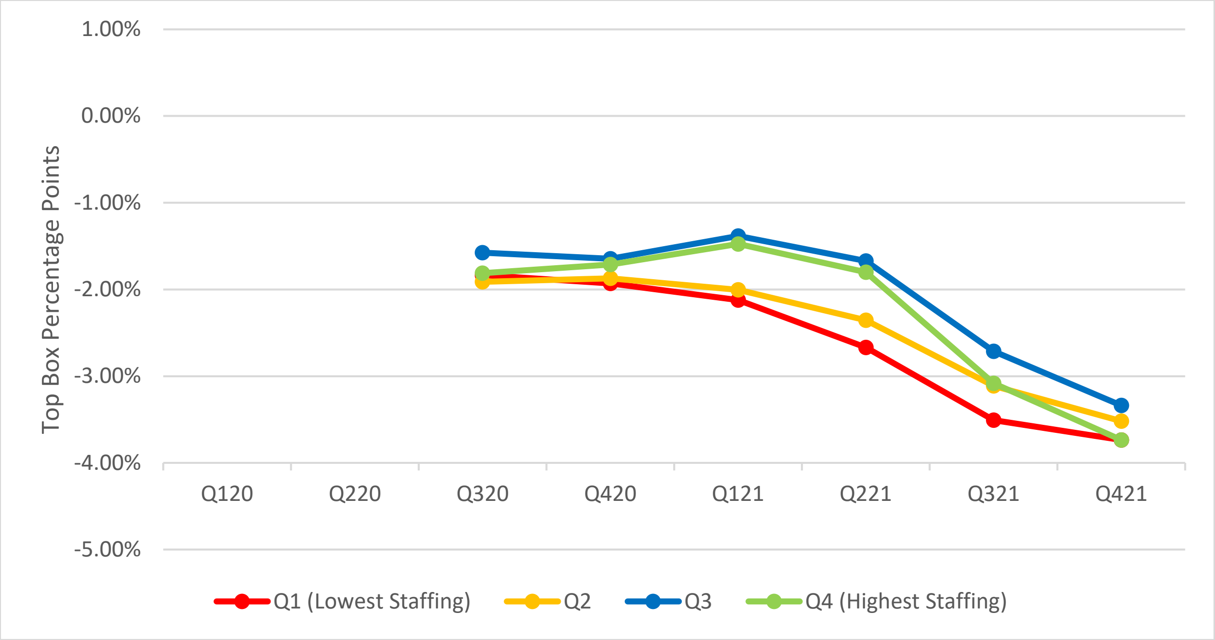

Note: Hospitals with higher 2019 staffing levels show generally smaller estimated pandemic effects on HCAHPS-SS when using the primary sample (as shown in Figure 3 and reproduced for easy comparison here as eFigure 4A) or either of the two constant cohorts (Alt 1 and Alt 2; eFigures 4B and 4C). Patterns do not appear to reflect differences between hospitals that voluntarily reported through the pandemic and other hospitals, nor do they appear to be a function of the entry and exit of hospitals over the study period.

Alt 1: the 1299 hospitals with at least 25 completed surveys in every quarter Q1/2018-Q4/2021 (voluntary reporters with sufficient sample size)
Alt 2: the 1402 hospitals with at least 25 completed surveys in all quarters Q1/2018-Q4/2019 plus Q3/2020-Q4/2021 (those with sufficient sample size, ignoring voluntary reporting).

© 2023 Elliott MN et al. *JAMA Health Forum.*

eFigure 5: Average Quarterly HCAHPS Hospital Eligible Discharges per Reporting Hospital, Q118-Q421

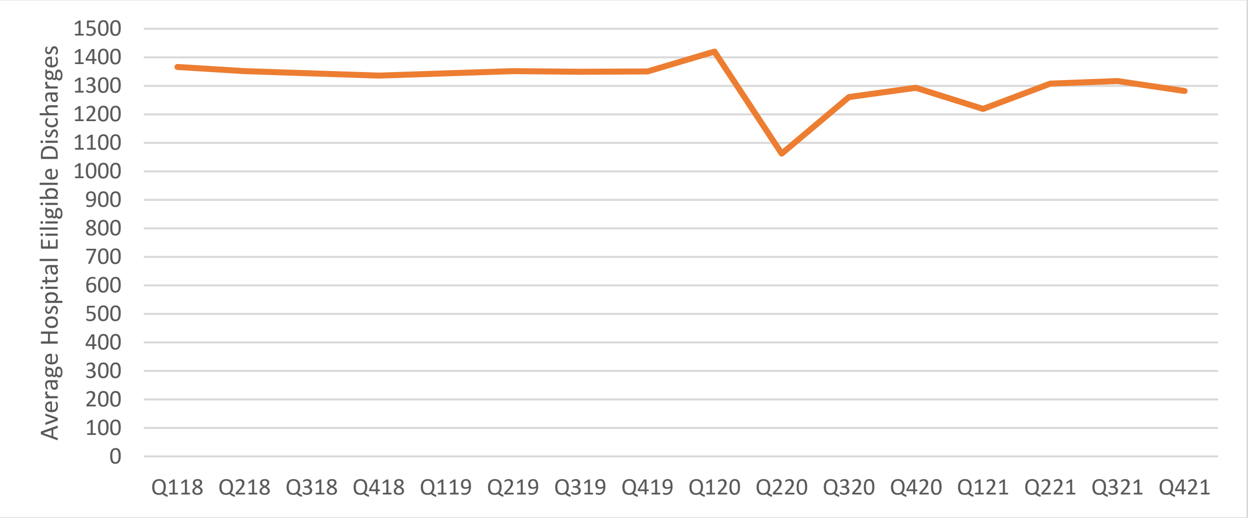

Note: Average HCHAPS-eligible patient volume was stable from Q1/2018 thru Q4/2019, followed by a 5.0% increase in Q1/2020 (compared to the mean of Q1/2018-Q4/2019) followed by a 21.3% decrease in Q2/2020 (also compared to the mean of Q1/2018-Q4/2019). Thereafter, volume mostly recovered to a level just below 2018-2019 volume: Q3/2020-Q4/2021 averaged 3.5% lower than Q1/2018-Q4/2019. These patterns do not correspond to changes in HCAHPS-SS in Figure 1 and eFigure 1.

© 2023 Elliott MN et al. *JAMA Health Forum.*

eFigure 6: Quarterly Estimates of Pandemic Effect on HCAHPS-SS by Census Division

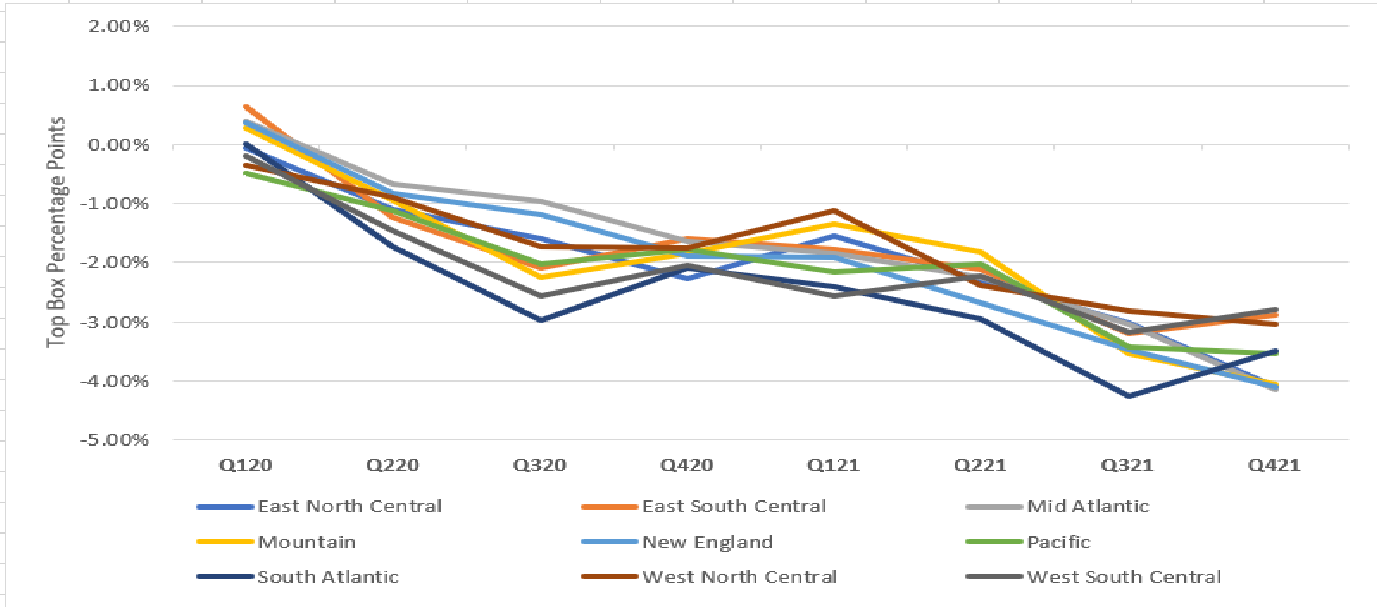

Also see eTables 4A-B

States in each Census Division: East North Central: IL, IN, MI, OH, WI; East South Central: AL, KY, MS, TN; Mid-Atlantic: NJ, NY, PA; Mountain: AZ, CO, ID, MT, NM, NV, UT, WY ; New England: CT, MA, ME, NH, RI, VT; Pacific: CA, OR, WA, HI, AK; South Atlantic: DC, DE, FL, GA, MD, NC, SC, VA, WV; West North Central: IA, KS, MN, MO, NE, ND, SD; West South Central: AR, LA, OK, TX.

© 2023 Elliott MN et al. *JAMA Health Forum.*
