# Supplementary material for: Changes in Patient Experiences of Hospital Care During the COVID-19 Pandemic
Source: JAMA Health Forum. 2023 Aug 25;4(8):e232766. doi: 10.1001/jamahealthforum.2023.2766 (PMC10457712; doi:10.1001/jamahealthforum.2023.2766)
Supplement: Supplement 2. — Data sharing statement [file jamahealthforum-e232766-s002.pdf]

Data Sharing Statement

Elliott. Changes in Patient Experiences of Hospital Care During the COVID-19 Pandemic. *JAMA Health Forum*. Published August 25, 2023. doi:10.1001/jamahealthforum.2023.2766

Data
Data available: No
